# A psychological model of competitive behavior: social comparison as a mediator of the critical thinking, self-efficacy, and adaptation ability prediction among college students

**DOI:** 10.1016/j.heliyon.2022.e12205

**Published:** 2022-12-10

**Authors:** M.M. Tri Susetyaning Mildawani, Tri Ratna Murti, Anastasia Sri Maryatmi, Juneman Abraham

**Affiliations:** a Management Department, Faculty of Economic and Business, Perbanas Institute, Jakarta 12940, Indonesia; b Psychology Department, Faculty of Psychology, University of Persada Indonesia YAI, Jakarta 10430, Indonesia; c Psychology Department, Faculty of Humanities, Bina Nusantara University, Jakarta 11480, Indonesia

**Keywords:** Competitive behavior, Critical thinking ability, Self-efficacy, Adaptation ability, Social comparison

## Abstract

This study is aimed to test a theoretical model of the critical thinking, self-efficacy, and adaptation ability prediction towards competitive behavior, with social comparison as a mediator. Participants comprised 334 active university students in Jakarta, the capital of Indonesia. This study applied a predictive-correlational design, with data obtained using questionnaires and analyzed with Structural Equation Modeling. The analysis indicated that the theoretical model corresponds to empirical data (*RMSEA* = 0.00, *NNFI* = 1.00) showing that sample data is an accurate representation of the data that would be present in the population (*RMSEA* < 0.05, *NNFI* > 0.90). However, critical thinking ability can not predict competitive behavior as mediated by social comparison. This present study provides a deeper understanding that competition is a complex behavior that involves the dimensions of a person's cognitive, self, and adaptive qualities, that cannot be separated from the social context.

## 1 Introduction

We live in the Industry 4.0 era, a period marked by increasing digitalization and connection as a result of physical digital interface, network, and data-processing technologies, as well as greater process integration, real-time information exchange, autonomy, and virtualization, which leads to improved productivity and flexibility to the point where mass personalization is made feasible (Culot et al., 2020). With the development of Industry 4.0 nowadays, which allows the development of creativity to be accelerated, the competition between individuals, between groups, and even between countries is becoming increasingly fierce. Therefore, it is urgent to understand the factors that can predict competitive behavior.

Various cutting-edge models of competitive behavior have been developed. For example, Li et al. (2022) developed a model of competitive behavior in social networks. Panjaitan et al. (2021) proved that at the business organization level, competitive behavior is influenced by knowledge creation, innovativeness, and network capability. Perceived competition acts as a mediator between economic inequality and social vigilance (Cheng et al., 2021). Childhood personality traits also predict attitudes towards future workplace competition (Tóth et al., 2022). These things indicate that the dynamics of competitive behavior have individual (self) and social dimensions. Since the research of Rothkopf (1983) and Vincent (1992), competitive behavior models – due to their central role in explaining performance in lots of settings (academic, business etc.) – have received good scholars’ attention.

In the Social Cognitive Theory, Bandura and National Institute of Mental Health (1986) elaborated on how behavior is inseparable from an individual's abilities to respond to his/her social environment. Such abilities encompass cognitive factors, self-efficacy, and outcome expectancies. An example of behavior in terms of responding to the social environment is the strong desire for self-actualization. The process of actualization encourages competition between individuals, which is an individual's desire to optimally appear as the best.

Competitive behavior is the behavior related to individuals' past accomplishments, which is done by comparing their self-competence with other people's competence (J. M. Houston et al., 2002). Social interactions in this dynamic technological era demands individuals to be able to behave competitively, considering that competition exists in all areas of life, such as the household, on campus, and at the workplace (Barnett and Casper, 2001).

Frankl (2014) asserted that essentially, individuals desire to survive. Efforts of survival result from an individual's self-reflection toward the dynamic experiences of life, as part of the effort to survive “as a winner”. The drive to win then becomes part of an individual's work behavior (Bartram, 2005). Whether conscious or not, in daily life every individual display work behavior in the form of competitiveness, in terms of competing to fulfill life necessities. Competitive behavior is also an individual's competence for self-development (Mudrack et al., 2012).

Although it is operatively difficult to define, competitive behavior is a variable that can be understood as a behavior to achieve a certain superior position with an aim for survival. Two indicators have been used by J. Houston et al. (2002) to describe competition, being competition enjoyment and competition conscientiousness. Competition enjoyment involves taking pleasure in competing, including enjoyment when facing the “opponent” while competing, and/or experiencing satisfaction when competing. Conversely, individuals who are conscientious in competing will avoid arguing with others, stay composed or reserved, or choose to avoid conflict altogether.

Indonesia is a country that prioritizes and upholds harmony in its society. This contrasts with Western societies that tend to be individualistic. The phenomenon that the authors have observed is that some people may be more competitive when they are temporarily residing in individualistic societies, but revert to the conscientious competition when they move to a collectivist community. For example, a person, *A*, exhibits strong competitive behavior while *A* resides in a community in one American state. *A*'s competitive behavior emerges when *A* can achieve personal goals (such as being able to finish studying in a certain period) and meet predetermined objectives. But then, although *A* survived in a society with strong individual characteristics, *A* displayed weaker competitive behavior upon returning to Indonesia that is distinctly communal. *A* may behave a certain way due to perceptions toward the social environment (Bandura & National Institute of Mental Health, 1986). As such, individuals who were initially competitive may become more compromising toward the behavior of individuals in a generally less competitive setting.

Additionally, Indonesia's competitiveness index is below Singapore, Malaysia, Brunei, and the Philippines. This ranking is based on the level of formal education, vocation, and international ranking of students. The Indonesian Education Index (Indeks Pendidikan Indonesia) is in the 7^th^ position in ASEAN with a score of 0.622 (as cited in Gerintya, 2019). Based on this reality, establishing competitiveness is highly needed in Indonesia, particularly in the current dynamic technological era.

Considering that the millennial generation predominates the workforce nowadays, the authors are interested in examining the factors that affect competitive behavior in Generation Y. Studies on competitive behavior in Generation Y are deemed important considering the 4.0 Industrial revolution. In addition to Generation Y predominating the workforce, they also comprise 50% of the population's productive subgroup. It is necessary to examine and study generation Y's potential, particularly concerning enhancing competitive behavior. According to Solnet and Hood (2008), The traits of Generation Y are distinctive, including the freedom to voice one's opinions, responsibility, and pleasure of challenges. Hornbostel et al. (2011) described that Generation Y desires environments that value contribution and talent. It can be said that one of Generation Y's distinct characteristics is that they enjoy challenges and competition.

Based on the state-of-the-art above, this study is aimed at testing the prediction of critical thinking ability, self-efficacy, and adaptation ability towards competitive behavior, as mediated by social comparison.

### 1.1 Theoretical review

Garcia et al. (2006) asserted that competitive behavior is an individual's behavior in attaining a certain superior position, as a manifestation of social comparison. In this study, social comparison is an individual's tendency to compare the opinions and competencies of other individuals, aimed at setting more objective quality standards (Garcia et al., 2013). An extensive series of studies by Garcia et al. (2006) discovered that the intensity of competition increases when individuals make comparisons with other individuals, who are termed competitors, and are able to position themselves at least equal to their competitors. Competitors in this regard are colleagues, or “companions in arms” who meet certain social categories (childhood peers, school peers, college peers from the same faculty or university). The aspects of social comparison used in this study are the perception of a certain standard, perception toward opinion comparisons, and perceptions of competence (Garcia et al., 2013).

How does the ability to think critically relate to competitive behavior? A cognitive psychologist expert, Chance (2013) defined critical thinking ability as the ability to analyze facts, elicit and arrange ideas, defend opinions, make comparisons, draw conclusions, evaluate arguments and solve problems. The individual factors behind critical thinking ability function as the bases for innovation in the effort to survive (McLeod, 2016). According to Tidd (2006), critical thinking ability is a variable that enables individuals to make innovations that directly contribute to an individual's achievements. With the critical thinking abilities they have, individuals will be able to optimize their rationality in achieving their goals, especially in demonstrating competitive behavior.

Critical thinking ability is defined as an individual's activity in terms of their capability to think analytically, synthetically, and solve problems (Kelloway et al., 2000). This ability is assumed to be the primary means of innovating necessarily (Damon et al., 2012). With their cognitive abilities, individuals are enabled to innovate in demonstrating their superiority, and the abilities they possess, in certain formulations (Chen et al., 2007). Aamodt (2010) asserted that critical thinking ability is an important intellectual capacity that plays a role in competing. Critical thinking ability is widely used as a factor that is predicted to be used in actualizing competence potential (Cherry and Ellis, 2005). The aspects used to measure critical thinking ability in this study are aspects proposed by Bandura (2001) and Damon et al. (2012), consisting of the ability to analyze, synthesize, and solve problems. **This present study hypothesizes that these abilities will be facilitated in social comparison, which leads to competitive behavior.**

In addition to cognitive abilities, competitive behavior may appear when individuals have self-efficacy and abilities to adapt to the dynamics that accompany competitive behavior.

Self-efficacy can predict competitive behavior (McClelland, as cited in Braden, 2000). Self-efficacy is part of an individual's capabilities, which is defined as the belief or conviction in personal abilities that enable individuals to overcome opportunities and obstacles (Schultz and Schultz, 2006). In his study, Bandura stated that self-efficacy is treated as one of the variables that affect competitive behavior (Bandura, 1997). Self-efficacy enables individuals to demonstrate and reinforce their competence in competing. Self-efficacy, therefore, serves as a variable to identify personal competence, to then regulate and manage it according to the situation at hand (Rodgers et al., 2014).

Self-efficacy is the ability to understand personal potential and abilities to then demonstrate it. Self-efficacy is a concept development from Bandura's first formulation of self-competence. Such efficacy is a predictive factor in competitive behavior (Bandura, 1994, 2005). Bandura (1994, 2005) proposed factors of self-efficacy include: the ability to optimize self-knowledge, the ability to self-regulate, and the ability to demonstrate personal competence. While according to Pajares and Schunk (2001), self-efficacy is the fundamental element that enables individuals to optimize the function of other capabilities, such as the ability to self-evaluate, self-regulate, and adapt.

Bandura (1994, 2005) incorporated social feedback as input to increase self-efficacy, not only self. For Bandura, vicarious experiences are a source of self-efficacy in addition to the other three sources (mastery experiences, emotional and physiological states, dan social persuasion). Related to this proposition, Bandura (1998, p. 626) stated, “Seeing people similar to oneself succeed by sustained effort raises observers' beliefs that they too possess the capabilities to master comparable activities to succeed. Modeling influences do more than a provide a social standard against which to judge one's own capabilities.”

Social comparison is an activity to obtain information about the success of others, which in this case can be a source of self-efficacy. **The present study hypothesizes that feelings toward self-efficacy will be facilitated in social comparison when demonstrating competitive behavior.**

Adaptation ability is an individual's ability to adjust to environmental demands so that individuals may present their selves (Senge, 2006). This adaptation ability is considered an individual's capacity to adjust and synchronize with environmental demands. It results from the reciprocal interaction between the individual and the social environment. In social life, individuals and the environment influence each other. This reciprocal interaction is the main framework of the Social Cognitive Theory (Bandura & National Institute of Mental Health, 1986). With their adaptive abilities, individuals become agents of change that can enhance their potential capacity, by activating cognition as a source of knowledge, self-recognition, and ability to self-evaluate, among others (Pajares and Schunk, 2001). Bartram (2005) asserted that this ability is needed when individuals face changes, particularly sudden changes that require immediate action. Within this adaptation ability lies skills to identify personal competence, skills to strategically present one's self, and the ability to prioritize adapting, along with its options.

This adaptation ability is operationalized through the individual's perceptions of competences, adaptation strategies, and adaptation priorities (Garcia and Tor, 2007). Adaptive abilities help facilitate the internalization of new values within a person, as moderated by certain cognitive abilities (McLeod, 2016). Competitive behavior is shown by individuals who are highly capable of establishing interpersonal interactions, and vice versa. In less competitive individuals, interpersonal skills are less apparent (J. Houston et al., 2002). **This present study hypothesizes that adaptive abilities will be facilitated in social comparisons when actualizing competitive behavior.**

This study's problem formulation is as follows:

Does the theoretical model of the Critical Thinking Ability, Self-Efficacy, and Adaptation Ability prediction towards Competitive Behavior with Social Comparison as a mediator fit the empirical data?

## 2 Materials & methods

The research subjects were active students at the Atma Jaya Catholic University of Indonesia's Faculty of Psychology, Jakarta, Indonesia. The subjects were all Generation Y, or those born between 1982-2000 (Meier et al., 2010) and were completing their education at higher learning institutes. Generation Y was selected as the participant in this study assuming that this generation adds to the workforce population every year (Bennett et al., 2012). These students neither want to have to compete in finishing their studies nor compete to secure a job, as statistics show that employment opportunities for Generation Y are scarce.

The active student population of the Faculty of Psychology at the time of sampling in the Even Semester of 2017 was 1157 people (PDDikti, 2018). By using the Sample size calculator (n.d.), it was found that with the criteria of Confidence Level 95% and Margin of Error 5%, the minimum number of samples required was 289 students.

Initially, the total amount of questionnaires distributed from 7 Mei to 7 June 2018 was 370 questionnaires as field tests. Data was collected through convenience sampling. Participants filled out written informed consent. In the data analysis, only 334 questionnaires were eligible for analysis (48 males, 286 females). While 36 (or 9.73%) were excluded from further analysis due to incomplete data. Data was obtained after official clearance based on a Research Statement Letter from Atma Jaya Catholic University of Indonesia's Faculty of Psychology No. 011AA/III/D.FP-PP.80.03/1/2019, and a statement letter from Universitas Persada Indonesia Y.A.I No. 048/SR/D/SSC-UPI Y.A.I/XI/2018. This study was approved by the institutional review board, letter No. 629/D/Fak.Psi./UPI Y.A.I/VIII/2019.

The number of female participants exceeded males because generally, psychology is undertaken mostly by female students (Clay, 2017).

Participants’ age groups are presented in Table 1.

**Table 1:** Description of participants’ age.

Age Range	Frequency	%
18	91	27
19–20	119	36
21–22	78	23
23–24	46	14
Total	334	100

### 2.1 Theoretical model

**Figure 1:**
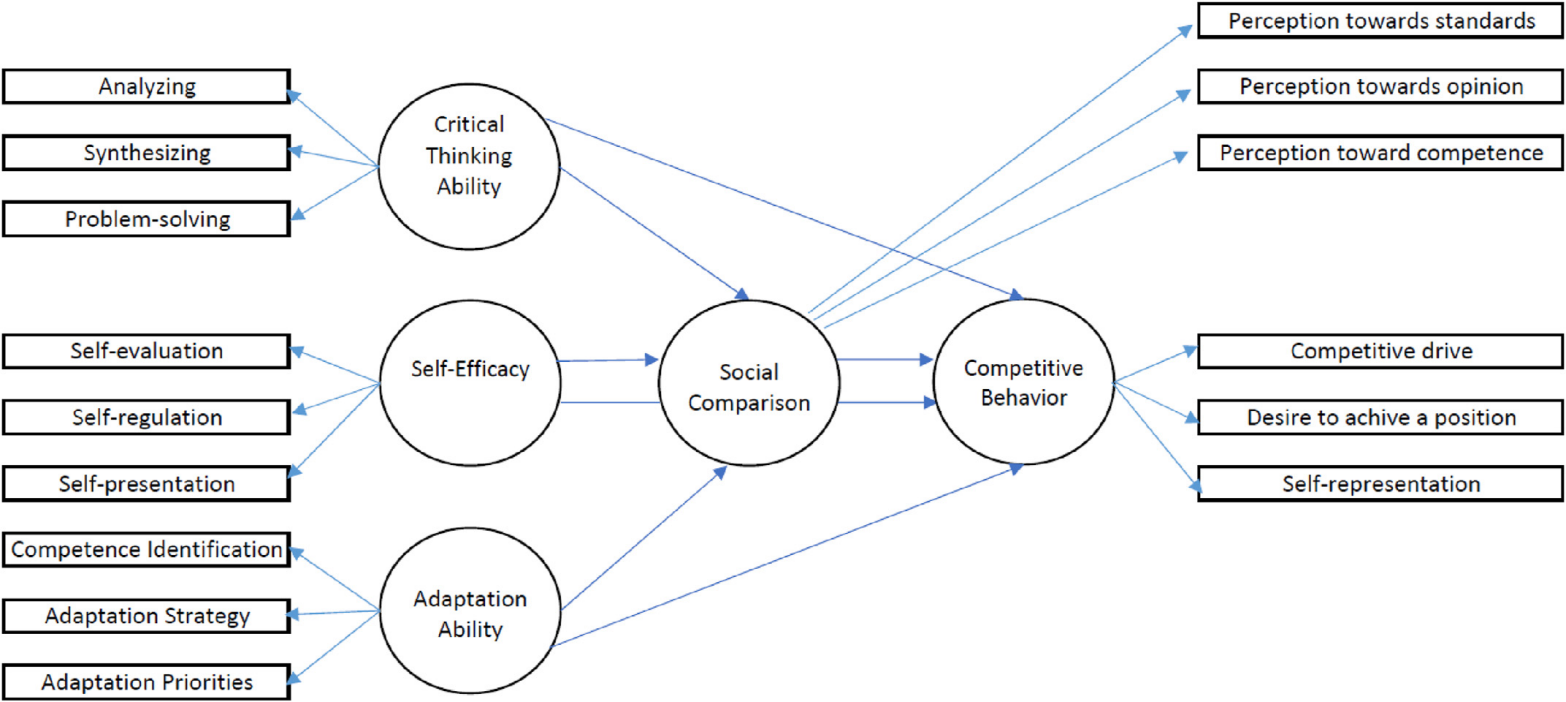
The predictive theoretical model of Critical Thinking Ability, Self-Efficacy, and Adaptation Ability towards Competitive Behavior with Social Comparison as a mediator.

The theoretical model is illustrated in Figure 1. In the model. competitive behavior is an endogenous variable. Critical thinking ability, self-efficacy, and adaptation ability are exogenous variables. Social comparison is a mediating variable.

### 2.2 Instruments

This study obtained data by using several instruments in questionnaire form, constructed on a 5-point Likert scale ranging from *Strongly Disagree* (scored 1) to *Strongly Agree* (scored 5).

In this study, Competitive Behavior is operatively defined as personal behavior to achieve a superior position with certain targets or standards that have been previously agreed upon. Competitive behavior is measured on a behavioral scale based on aspects of competitive behavior, such as the urge to compete, the desire to achieve superiority, and self-representation (Graf et al., 2012). This scale has 22 items in total, with 12 favorable items and 10 unfavorable items (reversely scored).

Item examples for each dimension are as follows:

• *On the urge to compete (competitive drive)* dimension: (1) I am a person who likes to compete; (2) My achievements are results of competing.
• *On the desire to achieve superiority (desire to achieve a position)* dimension: (1) I do not intend to be the best (unfavorable item); (2) I enjoy competing with peers.
• *On the self-representation* dimension: (1) I am determined to exceed my peers' accomplishments; (2) Competitors are needed to motivate us in studying.

In this study, Social Comparison is operatively defined as the tendency for individual behavior to evaluate self-competence by comparing their personal competence with the competence and opinions of others. Social comparison is measured on a scale constructed by the authors based on Garcia et al.’s (2013) aspects of Social Comparison, being perceptions toward certain standards, perceptions toward opinion comparisons, and perceptions toward other people's competence. This scale has 22 total items comprising 12 favorable items and 10 unfavorable items (reversely scored).

Item examples for each dimension are as follows:

• *On the perceptions toward standards* dimension: (1) Standards are needed to measure personal qualities; (2) Quality standards are needed to be an indicator of work results.
• *On the perceptions toward opinion comparisons* dimension: (1) I never think about other people's opinions, when I am setting a goal (unfavorable item); (2) I never involve other people's opinions when working (unfavorable item).
• *On the perceptions toward competence differences* dimension: (1) Comparing one's competence with a peer's competence, is necessary in order to work well; (2) I never compare my abilities with the abilities of my peers (unfavorable item).

In this study, Critical Thinking Ability is operationally defined as an individual's cognitive ability to process analytically, synthesize, and solve problems (Bandura, 2001). Critical thinking ability is measured on a scale based on these three aspects. This scale has 24 total items comprising 12 favorable items and 12 unfavorable items (reversely scored).

Item examples for each dimension are as follows:

• *On the analytical thinking* dimension: (1) The ability to analyze is fundamental to work quality; (2) Before completing a task, I must first understand the situation.
• *On the synthetic thinking* dimension: (1) To achieve objectives, then adequate knowledge is needed, and (2) Knowledge is the main key to finishing a job.
• *On the problem-solving* dimension: (1) In the current competitive era, then one must think strategically; (2) The ability to think strategically is key in completing a job.

In this study, Self-Efficacy is operatively defined as an individual's belief in their ability to self-regulate and demonstrate competencies that serve as their capital to achieve predetermined goals. Self-efficacy is measured with an attitude scale based on aspects of self-evaluation, self-regulation, and personal presentation (Bandura, 2001). This scale has 23 total items comprising 12 favorable items and 11 unfavorable items (reversely scored).

Item examples for each dimension are as follows:

• *On the self-evaluation* dimension: (1) When I make plans, I am determined that I can realize them; (2) When I set important objectives for myself, I rarely accomplish them (unfavorable item).
• *On the self-regulatio*n dimension: (1) When I try to learn something new, I will immediately give up if from the beginning I am unsuccessful (unfavorable item); (2) When unexpected problems occur, I continue to focus on working.
• *On the self-presentation* dimension: (1) When I want to meet someone, then I will immediately meet them; (2) I do not face difficulties in social friendships.

In this study, Adaptation Ability is operationally defined as an individual's fundamental ability in adjusting to the environment as well as finding ways for strategic self-representation. Adaptation ability is measured with an adaptation attitude scale based on Senge's (2006) theoretical aspects of adaptive abilities, being the individual's competence identification, adaptation strategies, and adaptation priorities. This scale has 16 total items comprising 10 favorable items and 6 unfavorable items.

Item examples for each dimension are as follows:

• *On the identification of individual competence* dimension: (1) When “in battle”, it is necessary to understand personal competence; (2) To me, it does not take long to work together in a team.
• *On the identification of adaptation strategy* dimension: (1) The situational changes that I face is a reason not to complete a task/job (unfavorable item); (2) Identifying the advantages of the work opponent, is the key to achieving at work.
• *On the identification of adaptation priority* dimension: (1) When I set objectives, I must be able to complete them; (2) It takes a long time for me to adjust to my peers' work rhythm/pace (unfavorable item).

### 2.3 Data analysis

The questionnaire's items were deemed comprehensible as evaluated by three experts. The three experts were assigned as raters who scored each item. The item's constructs in all scales were then tested by using 2^nd^ order confirmatory factor analysis (CFA) (2^nd^ Order CFA) (*N* = 60). The Goodness of Fit criteria of the construct validities used *RMSEA* ≤ 0.08 and GFI, NFI, NNFI, and CFI ≥0.90. Whereas reliability estimates using Cronbach's alpha coefficient (Alpha ≥0.60), Construct Reliability (*CR* ≥ 0.70), and Variance Extract (*VE* ≥ 0.50). Additionally, item selection was based on item discrimination with the criteria of Corrected Item-Total Correlations (CIT ≥0.30).

Following these steps, a full SEM model (*N* = 370) with the *λ* parameter test (loading factor/indicator coefficients) measurement on exogenous and endogenous models. The analysis measured *t*-values and structural equation coefficients, by testing whether the *t*-value would exceed 1.96.

## 3 Results

### 3.1 Validity and reliability tests

Construct validity and reliability testing of the Competitive Behavior scale on 60 tryout participants demonstrated Goodness of Fit (GOF) from a 2^nd^ order CFA (Confirmatory Factor Analysis), *RMSEA* = 0.074, *GFI* = 0.92, *NFI* = 0.97, *NNFI* = 0.97, *CFI* = 0.98, and Cronbach's Alpha = 0.811, Construct Reliability (*CR*) = 0.94, and Variance Extract (*VE*) = 0.55 (Mildawani et al., 2019). Results of item selection based on discrimination power yielded 14 items with high discrimination, with a Corrected Item-Total Correlation (CIT) range of 0.328–0.612.

Results of construct validity and reliability testing of the Social Comparison scale on 60 tryout participants demonstrated Goodness of Fit (GOF) from a 2^nd^ order CFA (Confirmatory Factor Analysis), *RMSEA* = 0.070, *GFI* = 0.93, *NFI* = 0.96, *NNFI* = 0.97, *CFI* = 0.98, and Cronbach's Alpha = 0.819, *CR* = 0.94, *VE* = 0.54 (Mildawani, 2021a). Results of item selection based on discrimination power yielded 15 items with high discrimination, with a Corrected Item-Total Correlation (CIT) range of 0.370–0.650.

Results of construct validity and reliability testing of the Critical Thinking scale on 60 tryout participants demonstrated Goodness of Fit (GOF) from a 2^nd^ order CFA (Confirmatory Factor Analysis), *RMSEA* = 0.082, *GFI* = 0.92, *NFI* = 0.97, *NNFI* = 0.97, *CFI* = 0.98, and Cronbach's Alpha = 0.892, *CR* = 0.96, *VE* = 0.60. Results of item selection based on discrimination power yielded 24 items with high discrimination, with a Corrected Item-Total Correlation (CIT) range of 0.321–0.750.

Construct validity and reliability testing of the Self-Efficacy scale on 60 tryout participants demonstrated Goodness of Fit (GOF) from a 2^nd^ order CFA (Confirmatory Factor Analysis), *RMSEA* = 0.082, *GFI* = 0.92, *NFI* = 0.95, *NNFI* = 0.96, *CFI* = 0.97, and Cronbach's Alpha = 0.894, *CR* = 0.95, *VE* = 0.45. Hatcher (1994) explained that Variance Extracted (*VE*) testing is conservative, therefore *VE* results of <0.5 were rejected. Results of item selection based on discrimination power yielded 23 items with high discrimination (no items were eliminated), with a Corrected Item-Total Correlation (CIT) range of 0.338–0.711.

Results of construct validity and reliability testing of the Adaptation Ability scale on 60 tryout participants demonstrated Goodness of Fit (GOF) from a 2^nd^ order CFA (Confirmatory Factor Analysis), *RMSEA* = 0.066, *GFI* = 0.93, *NFI* = 0.97, *NNFI* = 0.98, *CFI* = 0.98, and Cronbach's Alpha = 0.747, *CR* = 0.94, *VE* = 0.53 (Mildawani, 2021b). Results of item selection based on discrimination power yielded 11 items with high discrimination, with a Corrected Item-Total Correlation (CIT) range of 0.319–0.504.

### 3.2 Descriptive results

Table 2 shows that the empirical mean of all variables is above the hypothetical mean (3.00), with the highest empirical mean on critical thinking ability (*M* = 4.09). Table 3 shows the correlations between variables. It appears that Competitive Behavior has the strongest correlation with Social Comparison (*r* = 0.354, *p* < 0.001), and Competitive Behavior has no correlation with Critical Thinking Ability (*r* = 0.107, *p* > 0.05).

**Table 2:** Description of study data.

	*N*	Minimum	Maximum	*M*	*SD*
Competitive Behavior	334	1.14	5.00	3.4386	0.60132
Social Comparison	334	1.00	5.00	3.6678	0.51341
Critical Thinking Ability	334	1.27	5.00	4.0904	0.48132
Self-Efficacy	334	1.00	5.00	3.4878	0.49760
Adaptation ability	334	1.00	5.00	3.9608	0.45307

**Table 3:** Correlation matrix.

Variable		Competitive Behavior		Social Comparison		Critical Thinking Ability		Self-Efficacy		Adaptation Ability
Competitive Behavior	Pearson's *r*	—								
	*p*	—								
	Upper 95% CI	—								
	Lower 95% CI	—								
Social Comparison	Pearson's *r*	0.354	∗∗∗	—						
	*p*	0.000		—						
	Upper 95% CI	0.444		—						
	Lower 95% CI	0.256		—						
Critical Thinking Ability	Pearson's *r*	0.107		0.102		—				
	*p*	0.051		0.061		—				
	Upper 95% CI	0.212		0.208		—				
	Lower 95% CI	-0.000		-0.005		—				
Self-Efficacy	Pearson's *r*	0.173	∗∗	0.239	∗∗∗	-0.047		—		
	*p*	0.002		0.000		0.391		—		
	Upper 95% CI	0.275		0.338		0.061		—		
	Lower 95% CI	0.067		0.135		-0.154		—		
Adaptation Ability	Pearson's *r*	0.173	∗∗	0.195	∗∗∗	0.549	∗∗∗	0.153	∗∗	—
	*p*	0.002		0.000		0.000		0.005		—
	Upper 95% CI	0.275		0.296		0.619		0.257		—
	Lower 95% CI	0.067		0.089		0.469		0.047		—

∗ *p* < .05, ∗∗ *p* < .01, ∗∗∗ *p* < .001

### 3.3 Assumption check results

Due to the imbalance in the number of samples between male (*N* = 48) and female (*N* = 286), as acknowledged in the *Materials & Methods* section, this present study conducted a test of differences for all variables based on sex, to check assumption of no gender bias. As shown in Table 4, there are no sex differences in almost all (namely 4 out of 5) variables, i.e. Competitive Behavior, Social Comparison, Critical Thinking Ability, and Self-Efficacy (*p* of *t-*test >0.05). There is a sex difference only in one variable, i.e. Adaptation Ability variable. Thus this assumption check proves that there is less gender bias, and indicates that the gender imbalance of the participants has less impact on the interpretation of the research results.

**Table 4:** Test of differences based on sex (male vs. female).

Variables		Levene's Test for Equality of Variances		t-test for Equality of Means						
		*F*	*p*	*t*	*df*	*p*	*MD*	*SE*	95% *CI*	
									Lower	Upper
Competitive Behavior	Equal variances assumed	2.152	0.143	1.289	332	0.198	0.057	0.044	-0.030	0.145
	Equal variances not assumed			1.112	57.888	0.271	0.057	0.051	-0.046	0.160
Social Comparison	Equal variances assumed	9.628	0.002	1.835	332	0.067	0.072	0.039	-0.005	0.149
	Equal variances not assumed			1.387	54.281	0.171	0.072	0.052	-0.032	0.176
Critical Thinking Ability	Equal variances assumed	0.088	0.767	1.405	332	0.161	0.078	0.055	-0.031	0.186
	Equal variances not assumed			1.484	66.786	0.142	0.078	0.052	-0.027	0.182
Self-Efficacy	Equal variances assumed	3.597	0.059	-0.794	332	0.428	-0.031	0.039	-0.109	0.046
	Equal variances not assumed			-0.710	59.086	0.480	-0.031	0.044	-0.120	0.057
Adaptation Ability	Equal variances assumed	7.469	0.007	3.795	332	0.000	0.177	0.047	0.085	0.268
	Equal variances not assumed			3.218	57.325	0.002	0.177	0.055	0.067	0.286

*Note*. *MD* = Mean Difference, *SE* = Standard Error of Difference, *CI* = Confidence Interval of Difference.

**Figure 2:**
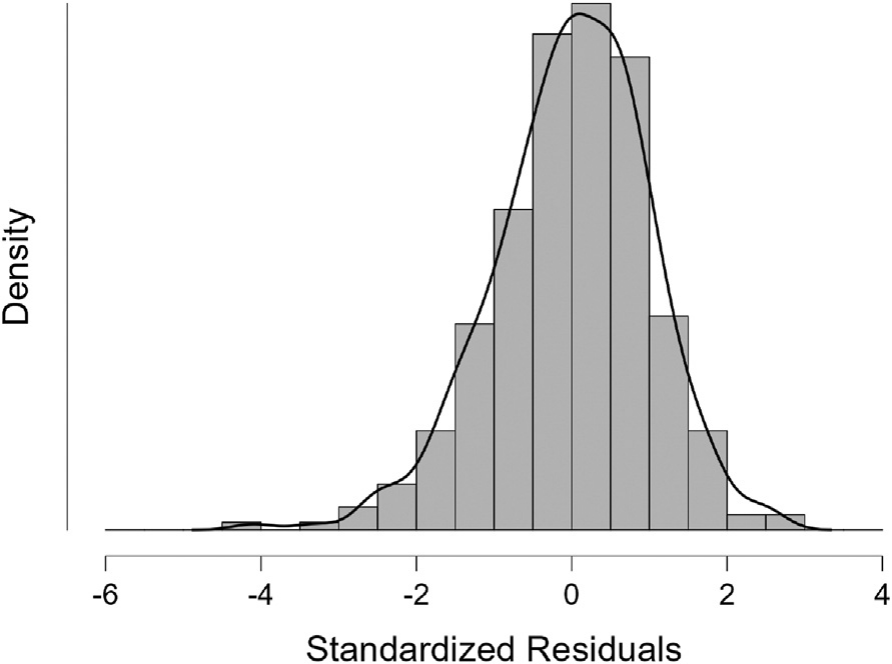
Results of normality test visualized as histogram. Source: JASP for Windows version 0.16.3 analysis results.

**Figure 3:**
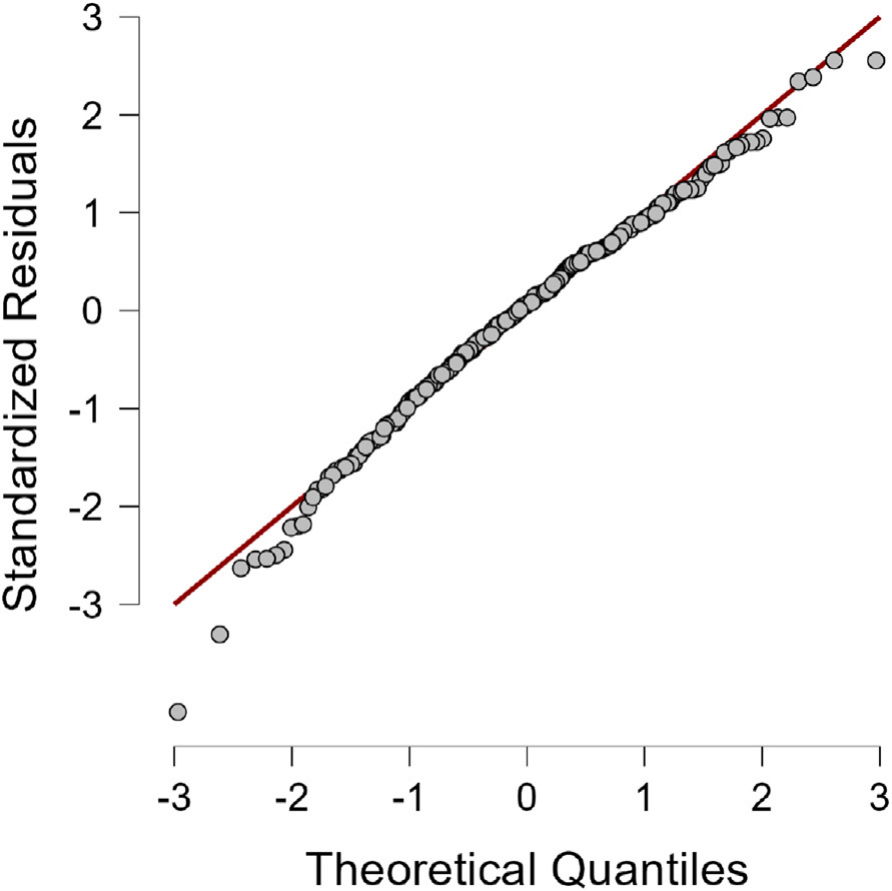
Results of normality test visualized as scatter plot. Source: JASP for Windows version 0.16.3 analysis results.

The next assumption check is the multivariate normality check. The residual normality test shows a relatively normal distribution. As shown in Figure 2, the ogive of the histogram produces a bell-like shape, and as shown in Figure 3, the Q-Q plot produces a scatter plot that follows the normal trend diagonal line. Both are evidence of multivariate normality. As shown in Table 5, the mean (*M*) and standard deviation (SD) of standardized residuals are -9.731 × 10^−4^ (or, closed to zero) and 1.003 (closed to 1) respectively. This is statistical support which indicates that there is no skewness, because the skewness of distribution means a lack of symmetry, while Figure 2 shows a relatively symmetrical distribution.

**Table 5:** Residual statistics.

	**Minimum**	**Maximum**	***M***	***SD***	***N***
Predicted Value	2.989	3.855	3.378	0.108	334
Residual	-1.081	0.674	9.323 × 10^−18^	0.264	334
Std. Predicted Value	-3.608	4.418	3.733 × 10^−16^	1.000	334
Std. Residual	-4.139	2.545	-9.731 × 10^−4^	1.003	334

### 3.4 Inferential results

Data was then processed using LISREL version 8.80 for Windows. A full SEM parameter λ (*loading factor*/indicator coefficient) testing model was done to measure both exogenous and endogenous models. This analysis measures t-values and structural equation models, by testing whether the t-value would exceed 1.96. This analysis was conducted to test if social comparison as a mediator in the role of critical thinking ability, self-efficacy, and adaptation ability, would fit empirical data.

**Figure 4:**
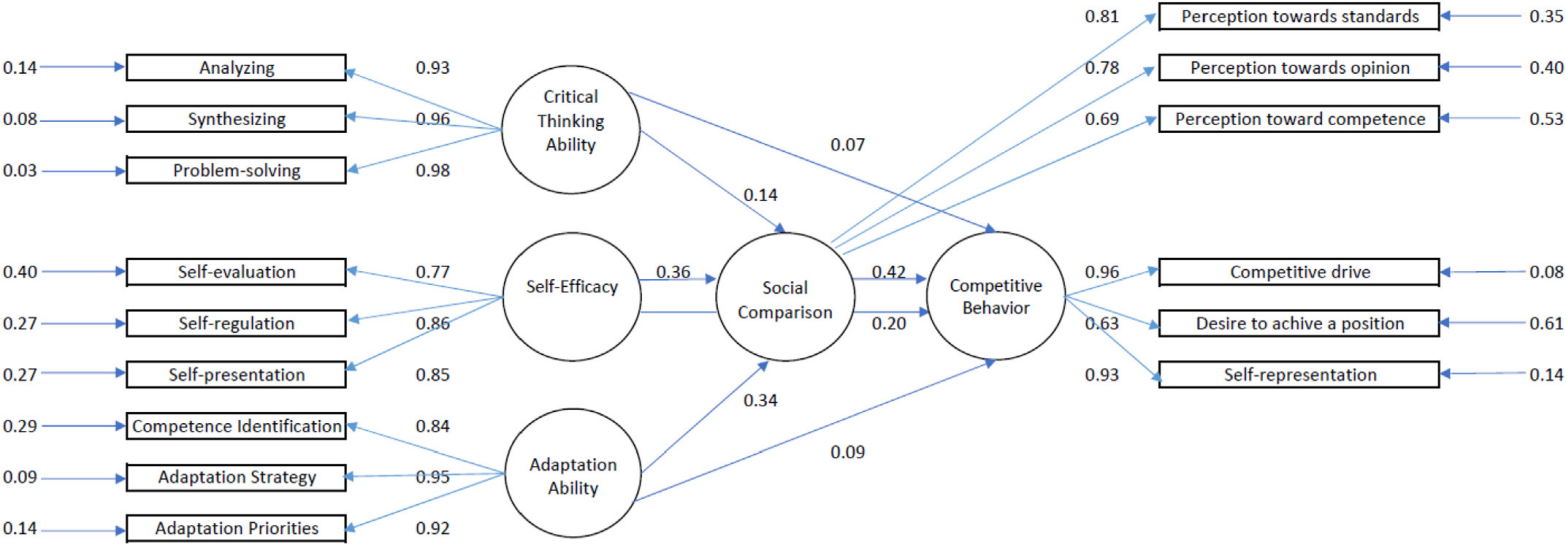
Structural model (standardized solutions).

**Figure 5:**
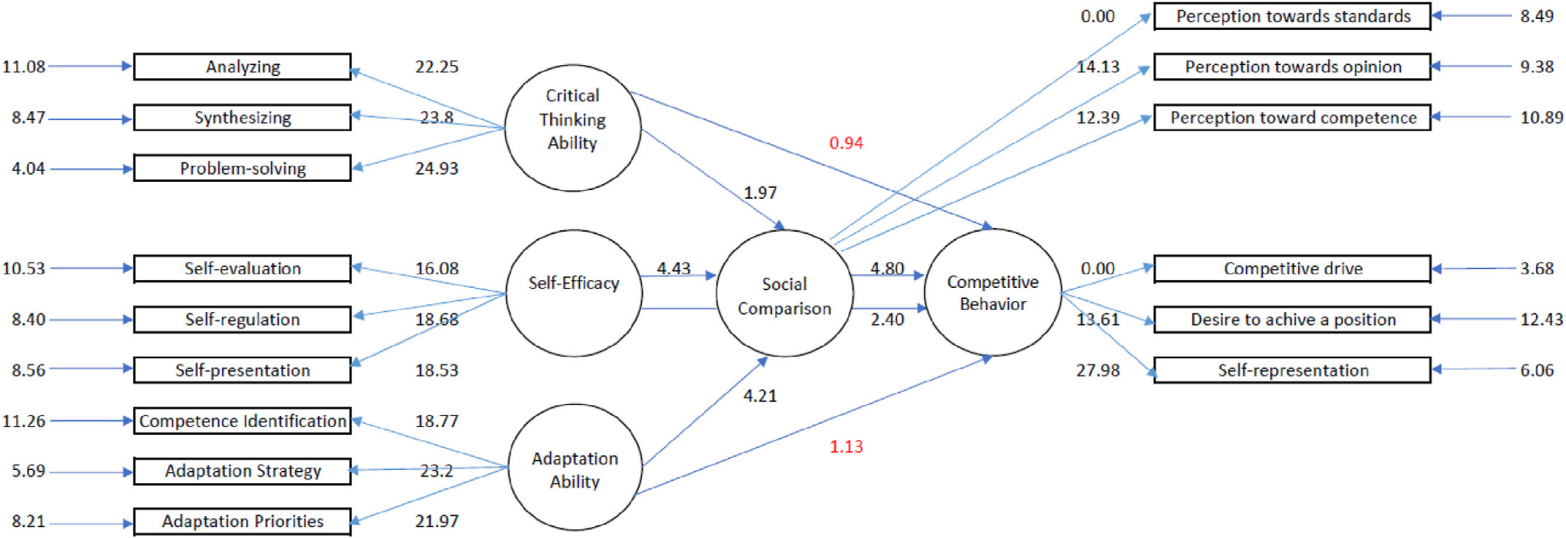
Structural model (T values).

The Goodness of Fit statistics are presented in Figures 4 and 5. Model testing toward the hypothetical model generated the following fit index: *Chi-square* = 25.95, *df* = 80, *p* = 1.00, *RMSEA* = 0.00, *GFI* = 0.99, *NFI* = 1.00, *NNFI* = 1.00, *CFI* = 1.00. The structural correlation between critical thinking ability, self-efficacy, and adaptation ability toward competitive behavior as mediated by social comparison, simultaneously generated an *R* coefficient of 0.94. A detailed presentation of this equation is shown in **Equation 1.** The results indicate that the proposed hypothesis on the predictive model of Critical Thinking Ability, Self-Efficacy, and Adaptation ability toward Competitive Behavior as mediated by Social Comparison, corresponded to empirical data.

The structural model illustrates a complete trajectory diagram with standardized numerical estimates.

*Note.* SC = Social Comparison, CTA = Critical Thinking Ability, SE = Self-Efficacy, AA = Adaptation Ability, CB = Competitive Behavior, Table 6 and Table 7 show coefficient values or parameters. These values have been previously estimated and used as a comparison of *t*-values to test the study's hypothesis.

**Table 6:** Evaluation of the structural model coefficient.

Path	Estimate	*t*	Statistical Significance
Social Comparison prediction towards Competitive Behavior	0.42	4.80 (*t* > 1.96)	Significant
Critical Thinking Ability prediction towards Competitive Behavior	0.07	0.94 (*t* < 1.96)	Insignificant
Self-Efficacy prediction towards Competitive Behavior	0.20	2.40 (*t* > 1.96)	Significant
Adaptation ability prediction towards Competitive Behavior	0.09	1.13 (*t* < 1.96)	Insignificant

**Table 7:** Analysis results of the direct and indirect estimate of critical thinking ability, self-efficacy, and adaptation ability toward competitive behavior through social comparison.

Path	Estimate			Statistical Significance
	Direct	Indirect	*t*	
Critical Thinking Ability – Competitive Behavior	0.07	0.06	0.94 (Direct) 1.82 (Indirect)	Insignificant (*t* < 1.96) Insignificant (*t* < 1.96)
Self-Efficacy – Competitive Behavior	0.20	0.15	2.40 (Direct) 3.36 (Indirect)	Significant (*t* > 1.96) Significant (*t* > 1.96)
Adaptation ability – Competitive Behavior	0.09	0.14	1.13 (Direct) 3.19 (Indirect)	Insignificant (*t* < 1.96) Significant (*t* > 1.96)

Based on the statistical significance in Table 6 and Table 7, it can be interpreted that (1) social comparison was able to predict competitive behavior, (2) self-efficacy was able to predict social comparison, (3) adaptation ability was able to predict social comparison, and (4) social comparison did not function as a mediator in the predictive relationship between critical thinking ability and competitive behavior.

## 4 Discussion

The results of this study address the problem formulation in the *Introduction* section, i.e. the theoretical model of the Critical Thinking Ability, Self-Efficacy, and Adaptation Ability prediction towards Competitive Behavior with Social Comparison as a mediator fits the empirical data. The following sections elaborate on the finding.

### 4.1 Social comparison predicts competitive behavior

This study found that **social comparison was able to predict competitive behavior**. The relationship between competitive behavior and social comparison has been substantiated since 1978 by Toda et al. (1978) in samples of children. Toda et al. (1978, p. 825) stated, “With increasing age, at least in the sample of American children observed, children learn competitive and cooperative choice rules that in appropriate settings serve long-term own-gain maximization.” Furthermore, recent research by Chen et al. (2021) showed that either actively comparing yourself to others, or passively being compared to others, can trigger competition.

Competitive behavior is learned along with chronological development (age). In Toda et al. (1978)'s experimental study, the social comparison condition is operationally defined as the condition whereby a child receives information on outcomes as retrieved from other children through a game, and the child receives such information repeatedly (more than once). It was found that such children become more competitive than children who receive information on such outcomes from their own doing.

Social comparison's predictive power toward competitive behavior appears to be consistent. Liu et al. (2021) found that social comparison orientation (SCO) – both SCO-ability and SCO-opinion – can predict trait competitiveness (TC) in a positive direction. According to them, this prediction can be understood because SCO and TC share the same features, namely based on centered on others and involving a comparison of the self to another person or people, either openly (SCO) or implicitly (TC).

Existing studies have even explored the relationship further in adolescents and adults, such as (1) conditions whereby social comparison produces healthy and unhealthy competitive behavior (note: not investigated in the present study), (2) differences in the intensity of competitive behavior between social comparisons that were made before, throughout, and after a competition, and (3) differences in competitive behavior based on social comparison as driven by individual and situational factors (Garcia et al., 2013, 2020). With regard to the consistent relationship between social comparison and competitive behavior, this study contributes to the anteceding factors of social comparison, being the ability to think critically, self-efficacy, and ability to adapt.

In this Industry 4.0, social comparison is ubiquitous due to online social media use (e.g. Facebook Instagram, TikTok). This symptom is labeled “Social Comparison 2.0” by Haferkamp and Krämer (2011). Social comparison in social media activities yields two outcomes, being (1) negative outcomes (e.g. envy, narcissistic behavior, a false sense of identity, psychological stress), or (2) positive outcomes (e.g. better mood management, learning about other people's positive behavior that are worth following) (Charoensukmongkol, 2018). An interesting dynamic to examine is social competition, which is the competition for attractiveness or dominance and power. One study applied a social rank perspective that stated that the intensity to which one feels superior or inferior to others and looked down on has a substantial impact on one's emotions and moods. Nonetheless, this study did not position competitive behavior as a positive conduct, unlike how the present study positions it.

Keupp et al. (2019, p. 4)'s analysis also showed a distinct feature of social comparison that humans tend to do, as stated in the following:

“Humans also compare themselves with others in the absence of direct competition, for example, in evaluative situations (which could be considered indirectly competitive) or out of social motivations such as conforming to group norms.”

Their findings showed that individuals make social comparisons in real competition situations and perceived competition. This finding reinforces the possibility of social competition in the context of online social networks, whereby competition tends to be imagined than direct. This present study does not differentiate between direct and indirect competition, or in other words, it encompasses both types of competition. Future studies may differentiate between the two to generate more precise information.

### 4.2 Social comparison predicted by self-efficacy before influencing competitive behavior

In discussing the function of social comparison as a mediator in the predictive relationship between self-efficacy and competitive behavior, then after discussing the relationship between social comparison and competitive behavior (discussed above under section *Social Comparison Predicts Competitive Behavior*), it is necessary to examine the association between self-efficacy and social comparison. This study found that **self-efficacy was able to predict social comparison.**

Self-efficacy and social comparison have been investigated repeatedly as two predictors of performance and productivity, whether singularly or in combination (Vrugt, 1994; Vrugt and Koenis, 2002). This is unsurprising as self-efficacy generates optimism and elicits one's best effort when dealing with obstacles and tasks, hesitance to achieve something, a greater desire to bring innovative solutions, which in turn will increase one's competence (Vrugt, 1994). Combined with social comparison, people with high self-efficacy will do “upward social comparison”, because they desire to “level up, model” or even “be better” than the target subjects of their social comparison (Vrugt, 1994).

Furthermore, Vrugt and Koenis (2002) asserted that self-efficacy is compatible with two out of three motives of social comparison, which is self-enhancement and self-improvement (the remaining motive is self-evaluation). In this Industry 4.0, the predictive relationship between social comparison and self-efficacy was confirmed in the context of online social games. Esteves et al. (2021) found that social comparison information in online games – namely achievement badges, leaderboards, level maps, and individual scores – was able to stimulate self-efficacy beliefs through vicarious experiences, which made an online social gamer have higher continuance behavior. After an online social gamer gains, exposure to the success of other gamers, that experience (upward social comparison) can increase his/her self-efficacy.

Nonetheless, not only in terms of upward social comparison but downward social comparison combined with self-efficacy is also able to generate positive feelings toward personal skills and competences, as comparing one's self with other people who have poorer performance may enhance one's self-esteem (Vrugt, 1994). It is evident that downward social comparison is compatible with one of three motives of social comparison, being self-evaluation (Vrugt and Koenis, 2002). People with high self-efficacy will maintain positive judgments toward their selves through downward social comparison; therefore one will also have higher perceived self-control. This means that such people will perceive their selves as having control over their performance, even in the face of setbacks or failure.

In an age where social media use is widespread, a potential issue that must be attended to is the emergence of negative emotions in social comparisons, particularly malicious envy and depressive emotions (Latif et al., 2021; Li, 2019). In this context, self-efficacy functions as psychological capital, whereby self-efficacy triggers positive coping styles, both cognitively and behaviorally, such as consistency and persistence in actions to achieve predetermined objectives (Li, 2019).

Based on the examination above, people with high self-efficacy need and possess higher preferences on social comparison, whether upward or downward. Self-efficacy may enhance motivation and escalation of efforts, and to optimize the results from one's exerted efforts, people with high self-efficacy require feedback that is facilitated through social comparison (Dissanayake et al., 2019).

### 4.3 Social comparison predicted by adaptation ability before influencing competitive behavior

In discussing the function of social comparison as a mediator in the predictive relationship between adaptation ability and competitive behavior, then after discussing the relationship between social comparison and competitive behavior (discussed above under section *Social Comparison Predicts Competitive Behavior*), it is necessary to examine the association between adaptation ability and social comparison. This study found that **adaptation ability was able to predict social comparison.**

Although Miao et al. (2018) found that social comparison predicts social adaptation, their explanation on this relationship may correspond to the present study's findings that, conversely, adaptation predicts social comparison. Adaptation is a behavior that supports survival needs, whereby in survival, individuals will exert various adaptation strategies. Miao et al. (2018) adopted Gilber et al.’s (1995, p. 2) presumption, that:

“From an evolutionary perspective, Gilbert et al. (1995) proposed that the need to compare oneself with others is very old in terms of phylogenetic development, biologically very powerful, and recognizable in many species, given the adaptive value of adequately sizing up one’s competitors.”

People with high adaptation abilities will use social comparison as part of adaptation strategies. Social comparison is viewed as the process to receive input, ideas, and feedback from others that build one's cognition and attention for survival (Miao et al., 2018). Social comparison facilitates the learning of the self, whether in terms of opinion or ability, attractiveness or intelligence, and unconsciously or consciously. In other words, doing social comparison is a form of responsibility in determining the meaningfulness of ourselves (Akter and Nweke, 2016). Meanwhile, life's meaningfulness is a necessary factor in the process of adaptation (Wong, 2012). Akter and Nweke (2016) added that in the Industry 4.0 context, social media is a “social environment for social comparison) (p. 474). Individuals with high adaptation abilities will utilize the social environment to achieve self-esteem and establish a “looking glass self in terms of social construction” (Akter and Nweke, 2016, p. 474). Self-growth is an adaptation process that relies on this “looking glass-self”, whereby individuals make personal reflections based on what others think and evaluate about them (Gecas & Schwalbe, as cited in Akter and Nweke, 2016).

The recent study of Boecker et al. (2022) showed that social comparison serves an adaptive function, namely balancing relationships and reducing inequality, by influencing a person's emotional and behavioral reactions to fortunes and misfortunes of other people or groups, both congruent (happy-for-ness, sympathy) and incongruent (schadenfreude, envy). With this latest insight, it is more understandable why people with higher adaptation abilities will also use social comparison in their daily lives.

### 4.4 Social comparison does not mediate the prediction of competitive behavior based on critical thinking ability

This study found that **social comparison did not function as a mediator in the predictive relationship between critical thinking ability and competitive behavior**. This may be due to the possibility that critical thinking requires collaboration or interdependence rather than competition. Long et al. (2022) suggested that critical thinking requires higher cognitive engagement in processing solid information and arguments, and either can be paradoxical or contradictive, and this can potentially be retrieved from peer collaboration rather than competition. Furthermore, Long et al. (2022) also explained that although social comparison may be epistemically motivating, or it may stimulate critical thinking particularly when the target comparison is a better model of critical thinking (a constructive effect), it also may cause concerns about being monitored or commented by the social environment, making critical thinking ineffective (a destructive effect). The existence of both constructive and destructive effects may cause a zero-sum game that causes social comparison to having no function as a mediator between critical thinking and competitive behavior.

In the learning context of Industry 4.0, Javadi et al. (2019) asserted that critical thinking requires a more conducive situation to converse, facilitated by online collaborative tools. Whereas in the context of competitive behavior, strategies to conceal certain information are considered necessary to win in more distinguished competitions (Hakim et al., 2021). When in fact, critical thinking is needed in a more open way and through shared information to be optimally processed.

## 5 Conclusions

Based on the study's testing of the theoretical model, it was concluded that (1) the theoretical model of critical thinking ability, self-efficacy, and adaptation ability prediction toward competitive behavior as mediated by social comparison, corresponds to empirical data; (2) social comparison positively predicts competitive behavior.; (3) critical thinking ability can not predict competitive behavior as mediated by social comparison; (4) self-efficacy positively influences competitive behavior as mediated by social comparison; and (5) adaptation ability positively predicts competitive behavior as mediated by social comparison.

This present study provides a novel contribution to developing a psychological model that combines elements of cognitive (critical thinking), self (self-efficacy, adaptation ability), and social (social comparison) in a parsimonious model.

The limitation is that it does not include affective elements that might contribute to an irrational competition. As studied by Kim and Wakslak (2013), irrational competition is a type of self-sacrificing competition. This deserves further research because it has no small impact in various fields, such as politics, economics, and education. The proposition of the school of psychoanalysis regarding human irrationality is still playing a role in today's competition. A “dry” cognitive approach may need to be complemented by an approach that considers the irrational side of humans. Therefore, to fully understand human competitive behavior, investigations into this aspect need to be expanded and deepened.

## Declarations

### Author contribution statement

M. M. Tri Susetyaning Mildawani: Conceived and designed the experiments; Performed the experiments; Analyzed and interpreted the data; Contributed reagents, materials, analysis tools or data; Wrote the paper.

Tri Ratna Murti; Anastasia Sri Maryatmi; Juneman Abraham: Conceived and designed the experiments; Analyzed and interpreted the data; Contributed reagents, materials, analysis tools or data; Wrote the paper.

### Funding statement

This research did not receive any specific grant from funding agencies in the public, commercial, or not-for-profit sectors.

### Data availability statement

Data will be made available on request.

### Declaration of interest's statement

The authors declare no conflict of interest.

### Additional information

No additional information is available for this paper.

## Appendix A Supplementary data

Questionnaire - revised.docx Questionnaire - Competitive Behavior and the Related Scales.docx
