# Supplementary material for: A psychological model of competitive behavior: social comparison as a mediator of the critical thinking, self-efficacy, and adaptation ability prediction among college students
Source: Heliyon. 2022 Dec 10;8(12):e12205. doi: 10.1016/j.heliyon.2022.e12205 (PMC9791351; doi:10.1016/j.heliyon.2022.e12205)
Supplement: Questionnaire - revised.docx [file mmc1.docx]

**Questionnaire Filling Instructions :**

1. **Here are some statements that need to be responded to, with some options.**
2. **Cross (X) on one option according to your condition.**
3. **There is no right or wrong answer, as long as the answer suits your condition.**
4. **Thank you for your cooperation.**

**STS : Very Not Suitable**

| **Description :** | | |
| --- | --- | --- |
| **SS** | **: Very Suitable** | **KS : Less Suitable** |
| **S** | **: Suitable** | **TS : Not Suitable** |

| **1** | **2** | **3** | **4** | **5** |
| --- | --- | --- | --- | --- |
| **STS**  **(Very Not Suitable)** | **TS**  **(Not Suitable)** | **KS**  **(Less Suitable)** | **S**  **(Suitable)** | **SS**  **(Very Suitable )** |

**Identity:**

Gender: Male/Female Date of birth:

Semester:

# 1. Competitive Behavior Scale

| **No.** | **Statement** | **STS** | **TS** | **KS** | **S** | **SS** |
| --- | --- | --- | --- | --- | --- | --- |
| 1 | I am a person who is challenged for the competition. |  |  |  |  |  |
| 2 | I enjoyed the atmosphere of the competition. |  |  |  |  |  |
| 3 | I'm not interested in achievements that are based on competition. |  |  |  |  |  |
| 4 | For me, friends are friends, not competitors. |  |  |  |  |  |
| 5 | I enjoyed the race to excel in the class. |  |  |  |  |  |
| 6 | When I was in class, I was encouraged to be the best. |  |  |  |  |  |
| 7 | Making friendships is more interesting than competing. |  |  |  |  |  |
| 8 | I don't like the atmosphere of ambitious competition. |  |  |  |  |  |
| 9 | I intend to be the best. |  |  |  |  |  |
| 10 | Excelling in class is more challenging to make than making friends. |  |  |  |  |  |
| 11 | Competition can reduce friendships. |  |  |  |  |  |
| 12 | I prefer friendship to competition. |  |  |  |  |  |
| 13 | I challenged to be the best when there are friends who are competitors. |  |  |  |  |  |
| 14 | Friends are good competitors. |  |  |  |  |  |
| 15 | For me, being a champion doesn't have to be in class. |  |  |  |  |  |
| 16 | The presence of a friend who became a competitor made me inferior in my work. |  |  |  |  |  |
| 17 | By being a champion, it is better known to people. |  |  |  |  |  |
| 18 | I have to be ahead of the competitors. |  |  |  |  |  |
| 19 | Instead of seizing, it is better to avoid competition. |  |  |  |  |  |
| 20 | I have the ambition to be the Class Leader . |  |  |  |  |  |
| 21 | I was challenged to be the Head of Organizational Affairs. |  |  |  |  |  |
| 22 | Defeating a friend who is a competitor is not a priority. |  |  |  |  |  |

**2. Social Comparison Scales**

| No. | Statement | **STS** | **TS** | **KS** | **S** | **SS** |
| --- | --- | --- | --- | --- | --- | --- |
| 1 | Competition can be conducted when there are certain standard criteria that are mutually agreed upon. |  |  |  |  |  |
| 2 | I don't pay attention to the standard of achievement in one particular area. |  |  |  |  |  |
| 3 | To maintain the quality of completion of lecture assignments, certain sizes / standards are needed. |  |  |  |  |  |
| 4 | So far, I don't care about the standards that are used as a reference for success. |  |  |  |  |  |
| 5 | Quality standards are needed to be used as a benchmark for work results. |  |  |  |  |  |
| 6 | Self-assessment can be used as a benchmark for success. |  |  |  |  |  |
| 7 | For me, a quality standard is necessary. |  |  |  |  |  |
| 8 | For me, other people's opinions are less relevant to be used as a standard in achievement. |  |  |  |  |  |
| 9 | When there is a standard to be achieved, I am challenged to achieve it. |  |  |  |  |  |
| 10 | Standards are not the only criteria that must be adhered to. |  |  |  |  |  |
| 11 | I am used to working with respect to existing standards. |  |  |  |  |  |
| 12 | Competitions can be conducted without targets. |  |  |  |  |  |
| 13 | I am used to working with certain targets. |  |  |  |  |  |
| 14 | Target, get me shackled at work. |  |  |  |  |  |
| 15 | I felt the need to compare the abilities I had with the abilities of other friends. |  |  |  |  |  |
| 16 | I am not affected by the performance of others. |  |  |  |  |  |
| 17 | The achievements of others become self-motivated. |  |  |  |  |  |
| 18 | I support the achievements of others. |  |  |  |  |  |
| 19 | I need the opinions of others, when setting one goal. |  |  |  |  |  |
| 20 | In this era of competition, it is necessary to compare self-ability with peer ability. |  |  |  |  |  |
| 21 | Comparison of self-competence with the competence of friends, needed to be able to work well. |  |  |  |  |  |
| 22 | There is no need to compare your abilities with the abilities of friends. |  |  |  |  |  |

1. **Critical** **Thinking Ability Scale**

| **No.** | **Statement** | **STS** | **TS** | **KS** | **S** | **SS** |
| --- | --- | --- | --- | --- | --- | --- |
| 1 | Logical thinking helps the completion of every job. |  |  |  |  |  |
| 2 | Analytical thinking is not the basis for working well. |  |  |  |  |  |
| 3 | With analytical skills, the work is easier to complete. |  |  |  |  |  |
| 4 | The ability to analyze is less necessary to work. |  |  |  |  |  |
| 5 | Analytical thinking skills are necessary to work well. |  |  |  |  |  |
| 6 | The ability to analyze is less helpful for job completion. |  |  |  |  |  |
| 7 | Analytical skills help logical thinking. |  |  |  |  |  |
| 8 | The success of a job does not depend on the analysis of thinking. |  |  |  |  |  |
| 9 | The success of work, determined by the ability of synthesis. |  |  |  |  |  |
| 10 | Synthesis ability is necessary for engineering work. |  |  |  |  |  |
| 11 | Synthesis work is the basis of success. |  |  |  |  |  |
| 12 | Success in work is determined by the ability to think synthesis. |  |  |  |  |  |
| 13 | To be able to achieve goals, the ability to think synthesis is needed. |  |  |  |  |  |
| 14 | Knowledge is the main key in getting the job done. |  |  |  |  |  |
| 15 | It takes synthesis thinking in completing each job. |  |  |  |  |  |
| 16 | Failure to compete due to inadequate knowledge. |  |  |  |  |  |
| 17 | In today's era of competition, one must think systematically. |  |  |  |  |  |
| 18 | When a person is lazy to think, then he will lose to compete. |  |  |  |  |  |
| 19 | Problems must be faced with logical thinking. |  |  |  |  |  |
| 20 | We should be able to analyze every problem at hand. |  |  |  |  |  |
| 21 | The ability to think systematically is the key to getting the job done. |  |  |  |  |  |
| 22 | I'm not used to working without thinking. |  |  |  |  |  |
| 23 | Usually, I am practical at work. |  |  |  |  |  |
| 24 | Work procedures are required at work. |  |  |  |  |  |

1. **Self-Efficacy Scale**

| No. | **Statement** | **STS** | **TS** | **KS** | **S** | **SS** |
| --- | --- | --- | --- | --- | --- | --- |
| 1 | When I make a plan, I believe I can realize it. |  |  |  |  |  |
| 2 | One of my problems is that I can’t work the way I have to. |  |  |  |  |  |
| 3 | If I can’t do the job, I keep trying until I can. |  |  |  |  |  |
| 4 | When I set important goals for myself, I rarely achieve them. |  |  |  |  |  |
| 5 | When I find it difficult at work, I give up before doing work. |  |  |  |  |  |
| 6 | When there is a difficulty, I avoid it. |  |  |  |  |  |
| 7 | If something looks complicated, I will still try it. |  |  |  |  |  |
| 8 | When there is something to be done, even if it is not fun to do, I will still solve it. |  |  |  |  |  |
| 9 | When I decide to do something, I will finish it. |  |  |  |  |  |
| 10 | When trying to learn something new, I will give up immediately if I don't succeed at first. |  |  |  |  |  |
| 11 | When unexpected problems occur, I continue to focus on working. |  |  |  |  |  |
| 12 | I avoid learning new things when it seems difficult to me. |  |  |  |  |  |
| 13 | Failure makes me try harder. |  |  |  |  |  |
| 14 | I feel unsure about my ability to do things. |  |  |  |  |  |
| 15 | I am an independent person. |  |  |  |  |  |
| 16 | I give up easily in the face of challenges at work. |  |  |  |  |  |
| 17 | I can't handle most of the problems that arise in life. |  |  |  |  |  |
| 18 | It's hard for me to make new friends. |  |  |  |  |  |
| 19 | If I want to meet someone, then I will meet that person. |  |  |  |  |  |
| 20 | If I meet someone who is interesting but difficult to make friends with, then I will not be friends with that person. |  |  |  |  |  |
| 21 | I still try to be friends with someone who seems unattractive. |  |  |  |  |  |
| 22 | I have no difficulty in social friendships. |  |  |  |  |  |
| 23 | I have a lot of friends. |  |  |  |  |  |

# Adaptation Ability Scale

| No. | **Statement** | **STS** | **TS** | **KS** | **S** | **SS** |
| --- | --- | --- | --- | --- | --- | --- |
| 1 | One of the keys to success is the ability to adapt to the environment. |  |  |  |  |  |
| 2 | I'm used to the establishment. |  |  |  |  |  |
| 3 | I am used to facing changes. |  |  |  |  |  |
| 4 | I was confused when I had to deal with a new challenge. |  |  |  |  |  |
| 5 | Problems at work must be resolved. |  |  |  |  |  |
| 6 | When I have to deal with new things, I can handle them. |  |  |  |  |  |
| 7 | The changing demands of the work must be resolved. |  |  |  |  |  |
| 8 | The sudden change of schedule made me "die of style". |  |  |  |  |  |
| 9 | I was able to deal with a changing situation. |  |  |  |  |  |
| 10 | Knowing the advantages of work opponents, is the key to achieving work performance. |  |  |  |  |  |
| 11 | Certain strategies are needed to deal with challenges. |  |  |  |  |  |
| 12 | I feel defeated when I have to struggle to get grades. |  |  |  |  |  |
| 13 | I'm used to working with erratic schedule. |  |  |  |  |  |
| 14 | For me, the sudden change is not a problem. |  |  |  |  |  |
| 15 | Planning is not important at work. |  |  |  |  |  |
| 16 | I find it hard to accept that situation without planning. |  |  |  |  |  |

**Indonesian Version**

**Petunjuk Pengisian Kuesioner:**

1. Isilah data identitas di atas, sesuai dengan kondisi anda.

2. Berikut ada beberapa pernyataan yang perlu direspon, dengan beberapa opsi pilihan.

3. Berilah tanda silang (X) pada satu opsi pilihan sesuai dengan kondisi anda.

4. Tidak ada jawaban yang benar atau salah, sejauh jawaban tersebut sesuai dengan kondisi anda.

5. Terima kasih atas kerjasama anda sekalian.

**STS : Sangat Tidak Sesuai**

| **Keterangan :** | | |
| --- | --- | --- |
| **SS** | **: Sangat Sesuai** | **KS : Kurang Sesuai** |
| **S** | **: Sesuai** | **TS : Tidak Sesuai** |

| **1** | **2** | **3** | **4** | **5** |
| --- | --- | --- | --- | --- |
| **STS**  **(Sangat Tidak Sesuai)** | **TS**  **(Tidak Sesuai)** | **KS**  **(Kurang Sesuai)** | **S**  **(Sesuai)** | **STS**  **(Sangat Tidak Sesuai)** |

**Identitas diri :**

Jenis kelamin: Laki-laki/Perempuan Tanggal Lahir:

Semester:

# 1. Skala Perilaku Kompetitif

| **No.** | **Pernyataan** | **STS** | **TS** | **KS** | **S** | **SS** |
| --- | --- | --- | --- | --- | --- | --- |
| 1 | Saya orang yang tertantang untuk kompetisi |  |  |  |  |  |
| 2 | Saya menikmati suasana kompetisi. |  |  |  |  |  |
| 3 | Saya tidak tertarik dengan prestasi yang didasarkan pada kompetisi. |  |  |  |  |  |
| 4 | Bagi saya, teman adalah sahabat, bukan kompetitor. |  |  |  |  |  |
| 5 | Saya menikmati perlombaan berprestasi di kelas, |  |  |  |  |  |
| 6 | Saat di kelas, saya terpacu untuk menjadi yang terbaik. |  |  |  |  |  |
| 7 | Menjalin persahabatan lebih menarik daripada berkompetisi. |  |  |  |  |  |
| 8 | Saya tidak menyukai suasana kompetisi yang ambisius. |  |  |  |  |  |
| 9 | Saya berniat untuk menjadi yang terbaik. |  |  |  |  |  |
| 10 | Berprestasi di kelas, lebih menantang untuk dilakukan daripada menjalin pertemanan. |  |  |  |  |  |
| 11 | Kompetisi dapat mengurangi pertemanan |  |  |  |  |  |
| 12 | Saya lebih memilih pertemanan daripada persaingan. |  |  |  |  |  |
| 13 | Saya tertantang menjadi yang terbaik ketika ada teman yang menjadi kompetitor. |  |  |  |  |  |
| 14 | Kawan adalah kompetitor yang baik. |  |  |  |  |  |
| 15 | Bagi saya, menjadi juara tidak harus di kelas. |  |  |  |  |  |
| 16 | Kehadiran kawan yang menjadi kompetitior, membuatku minder dalam berkarya. |  |  |  |  |  |
| 17 | Dengan menjadi juara, maka lebih dikenal orang. |  |  |  |  |  |
| 18 | Saya harus lebih unggul dari kompetitor. |  |  |  |  |  |
| 19 | Daripada rebut, lebih baik menghindari kompetisi. |  |  |  |  |  |
| 20 | Saya berambisi menjadi Ketua Kelas. |  |  |  |  |  |
| 21 | Saya tertantang menjadi Ketua Bidang Organisasi. |  |  |  |  |  |
| 22 | Mengalahkan kawan yang menjadi kompetitor bukanlah prioritas. |  |  |  |  |  |

**2 Skala Perbandingan Sosial**

| No. | Pernyataan | **STS** | **TS** | **KS** | **S** | **SS** |
| --- | --- | --- | --- | --- | --- | --- |
| 1 | Kompetisi dapat dilakukan ketika ada kriteria standar tertentu yang disepakati bersama. |  |  |  |  |  |
| 2 | Saya tidak memperhatikan standar prestasi di satu bidang tertentu. |  |  |  |  |  |
| 3 | Untuk menjaga kualitas penyelesaian tugas perkuliahan, diperlukan ukuran/standar tertentu. |  |  |  |  |  |
| 4 | Selama ini, saya tidak peduli dengan standar yang dijadikan acuan keberhasilan. |  |  |  |  |  |
| 5 | Standar yang bermutu diperlukan untuk dijadikan patokan hasil pekerjaan. |  |  |  |  |  |
| 6 | Penilaian diri dapat dijadikan patokan keberhasilan. |  |  |  |  |  |
| 7 | Bagi saya, standar yang bermutu, diperlukan. |  |  |  |  |  |
| 8 | Bagi saya, opini orang lain kurang relevan untuk dijadikan standar dalam berprestasi. |  |  |  |  |  |
| 9 | Ketika ada standar yang harus dicapai, saya tertantang untuk meraihnya. |  |  |  |  |  |
| 10 | Standar bukan satu-satunya kriteria yang harus dipatuhi. |  |  |  |  |  |
| 11 | Saya terbiasa bekerja dengan memperhatikan standard yang ada. |  |  |  |  |  |
| 12 | Kompetisi dapat dilakukan tanpa target. |  |  |  |  |  |
| 13 | Saya terbiasa bekerja dengan target tertentu. |  |  |  |  |  |
| 14 | Target, membuatku terbelenggu dalam bekerja. |  |  |  |  |  |
| 15 | Saya merasa perlu membandingkan kemampuan yang saya miliki dengan kemampuan teman lain. |  |  |  |  |  |
| 16 | Saya tidak terpengaruh dengan prestasi orang lain. |  |  |  |  |  |
| 17 | Prestasi orang lain menjadi motivasi diri. |  |  |  |  |  |
| 18 | Saya mendukung prestasi orang lain. |  |  |  |  |  |
| 19 | Saya membutuhkan pendapat orang lain, ketika menetapkan satu tujuan. |  |  |  |  |  |
| 20 | Di era persaingan ini, diperlukan perbandingan kemampuan diri dengan kemampuan rekan sebaya. |  |  |  |  |  |
| 21 | Perbandingan kompetensi diri dengan kompetensi teman, diperlukan untuk dapat bekerja dengan baik. |  |  |  |  |  |
| 22 | Tidak perlu membanding-bandingkan kemampuan diri dengan kemampuan teman. |  |  |  |  |  |

**3. Skala Kemampuan Berpikir Kritis**

| **No.** | **Pernyataan** | **STS** | **TS** | **KS** | **S** | **SS** |
| --- | --- | --- | --- | --- | --- | --- |
| 1 | Berpikir logis itu membantu penyelesaian setiap pekerjaan. |  |  |  |  |  |
| 2 | Berpikir analitis bukanlah dasar untuk dapat bekerja dengan baik. |  |  |  |  |  |
| 3 | Dengan kemampuan analisa, pekerjaan lebih mudah diselesaikan. |  |  |  |  |  |
| 4 | Kemampuan menganalisa kurang diperlukan untuk bekerja. |  |  |  |  |  |
| 5 | Kemampuan berpikir analitis diperlukan untuk bekerja dengan baik. |  |  |  |  |  |
| 6 | Kemampuan menganalisa kurang membantu penyelesaian pekerjaan. |  |  |  |  |  |
| 7 | Kemampuan analitis membantu logika berpikir. |  |  |  |  |  |
| 8 | Keberhasilan sebuah pekerjaan tidak tergantung pada analisa berpikir. |  |  |  |  |  |
| 9 | Keberhasilan kerja, ditentukan oleh kemampuan sintesis. |  |  |  |  |  |
| 10 | Kemampuan sintesis diperlukan untuk pekerjaan teknik. |  |  |  |  |  |
| 11 | Bekerja sintesis adalah dasar keberhasilan. |  |  |  |  |  |
| 12 | Keberhasilan dalam bekerja ditentukan oleh kemampuan berpikir sintesa. |  |  |  |  |  |
| 13 | Untuk dapat meraih tujuan, maka diperlukan kemampuan berpikir sintesis. |  |  |  |  |  |
| 14 | Pengetahuan adalah kunci utama dalam menyelesaikan pekerjaan. |  |  |  |  |  |
| 15 | Diperlukan berpikir sintesis dalam menyelesaikan setiap pekerjaan. |  |  |  |  |  |
| 16 | Kegagalan berkompetisi karena pengetahuan yang kurang memadai. |  |  |  |  |  |
| 17 | Di era kompetisi sekarang ini, maka seseorang harus berpikir sistematis. |  |  |  |  |  |
| 18 | Ketika seseorang malas berpikir, maka dia akan kalah bersaing. |  |  |  |  |  |
| 19 | Masalah harus dihadapi dengan berpikir logis. |  |  |  |  |  |
| 20 | Kita hendaknya melakukan analisa dalam setiap permasalahan yang dihadapi. |  |  |  |  |  |
| 21 | Kemampuan berpikir sistematis adalah kunci untuk menyelesaikan pekerjaan. |  |  |  |  |  |
| 22 | Saya tidak terbiasa bekerja tanpa berpikir. |  |  |  |  |  |
| 23 | Biasanya, saya praktis dalam bekerja. |  |  |  |  |  |
| 24 | Prosedur kerja diperlukan dalam bekerja. |  |  |  |  |  |

**4. Skala Efikasi Diri**

| No. | **Pernyataan** | **STS** | **TS** | **KS** | **S** | **SS** |
| --- | --- | --- | --- | --- | --- | --- |
| 1 | Ketika saya membuat rencana, saya yakin saya dapat merealisasikannya. |  |  |  |  |  |
| 2 | Salah satu masalah saya adalah saya tidak bisa bekerja sebagaimana mestinya. |  |  |  |  |  |
| 3 | Jika saya tidak dapat melakukan pekerjaan, saya terus mencoba sampai saya bisa. |  |  |  |  |  |
| 4 | Ketika saya menetapkan tujuan-tujuan penting untuk diri saya sendiri, saya jarang mencapainya. |  |  |  |  |  |
| 5 | Ketika saya merasa kesulitan saat bekerja, saya menyerah sebelum mengerjakan pekerjaan. |  |  |  |  |  |
| 6 | Ketika ada kesulitan, saya menghindarinya. |  |  |  |  |  |
| 7 | Jika sesuatu terlihat rumit, saya tetap akan mencobanya. |  |  |  |  |  |
| 8 | Ketika ada sesuatu yang harus diselesaikan, walaupun tidak menyenangkan untuk dilakukan, saya tetap akan menyelesaikannya. |  |  |  |  |  |
| 9 | Ketika saya memutuskan untuk melakukan sesuatu, saya akan menuntaskannya. |  |  |  |  |  |
| 10 | Ketika mencoba untuk mempelajari sesuatu yang baru, saya akan segera menyerah jika pada awalnya saya tidak berhasil. |  |  |  |  |  |
| 11 | Ketika masalah tak terduga terjadi, saya terus fokus bekerja. |  |  |  |  |  |
| 12 | Saya menghindari belajar hal-hal baru ketika terlihat sulit bagi saya. |  |  |  |  |  |
| 13 | Kegagalan membuat saya berusaha lebih keras. |  |  |  |  |  |
| 14 | Saya merasa tidak yakin tentang kemampuan saya untuk melakukan sesuatu. |  |  |  |  |  |
| 15 | Saya adalah orang yang mandiri. |  |  |  |  |  |
| 16 | Saya mudah menyerah dalam menghadapi tantangan dalam pekerjaan. |  |  |  |  |  |
| 17 | Saya tidak mampu menangani sebagian besar masalah yang muncul dalam kehidupan. |  |  |  |  |  |
| 18 | Sulit bagi saya untuk mendapat teman baru. |  |  |  |  |  |
| 19 | Jika saya ingin bertemu dengan seseorang, maka saya akan menemui orang tersebut. |  |  |  |  |  |
| 20 | Jika saya bertemu seseorang yang menarik tetapi sulit berteman, maka saya tidak akan berteman dengan orang itu. |  |  |  |  |  |
| 21 | Saya tetap mencoba berteman dengan seseorang yang tampaknya tidak menarik. |  |  |  |  |  |
| 22 | Saya tidak mengalami kesulitan dalam pertemanan sosial. |  |  |  |  |  |
| 23 | Saya mempunyai banyak teman. |  |  |  |  |  |

# 5. Skala Kemampuan Beradaptasi

| No. | **Pernyataan** | **STS** | **TS** | **KS** | **S** | **SS** |
| --- | --- | --- | --- | --- | --- | --- |
| 1 | Salah satu kunci keberhasilan adalah kemampuan adaptasi dengan lingkungan. |  |  |  |  |  |
| 2 | Saya terbiasa dengan kemapanan. |  |  |  |  |  |
| 3 | Saya terbiasa menghadapi perubahan-perubahan. |  |  |  |  |  |
| 4 | Saya bingung ketika harus berhadapan dengan tantangan baru. |  |  |  |  |  |
| 5 | Masalah dalam bekerja harus dibereskan. |  |  |  |  |  |
| 6 | Ketika harus menghadapi hal-hal baru, saya mampu mengatasinya. |  |  |  |  |  |
| 7 | Tuntutan pekerjaan yang berubah, harus diselesaikan. |  |  |  |  |  |
| 8 | Perubahan skedul/jadwal yang mendadak membuatku “mati gaya”. |  |  |  |  |  |
| 9 | Saya mampu menghadapi situasi yang berubah- ubah. |  |  |  |  |  |
| 10 | Mengetahui kelebihan lawan kerja, adalah kunci untuk meraih prestasi kerja. |  |  |  |  |  |
| 11 | Dibutuhkan strategi-strategi tertentu untuk menghadapi tantangan. |  |  |  |  |  |
| 12 | Saya merasa kalah ketika harus bersusah payah meraih nilai. |  |  |  |  |  |
| 13 | Saya terbiasa bekerja dengan skedul/jadwal yang tidak menentu. |  |  |  |  |  |
| 14 | Bagi saya, perubahan yang mendadak itu bukan masalah. |  |  |  |  |  |
| 15 | Perencanaan bukanlah hal yang penting dalam bekerja. |  |  |  |  |  |
| 16 | Saya sulit menerima situasi yang tanpa perencanaan. |  |  |  |  |  |
